# Children Learn Best From Their Peers: The Crucial Role of Input From Other Children in Language Development

**DOI:** 10.1162/opmi_a_00198

**Published:** 2025-04-29

**Authors:** Johanna Schick, Sabine Stoll

**Affiliations:** Institute for the Interdisciplinary Study of Language Evolution, University of Zurich, Zurich, Switzerland

**Keywords:** language development, input, child-surrounding speech, child-directed speech

## Abstract

Language input is crucial for language learning, with child-directed speech being a strong predictor of language development. Yet, in many non-industrialized rural societies, children are less exposed to this type of input. Instead, children encounter frequent child-surrounding speech from third-party interactions. Little is known about whether and how children learn language from this type of input. By analyzing naturalistic data from children growing up in the Shipibo-Konibo community in the Peruvian Amazon, we demonstrate that despite a high prevalence of child-surrounding input, child-directed input best predicts children’s production patterns defined as unigrams. We provide first evidence for remarkable similarities between child-surrounding speech and children’s own speech patterns. In addition, we demonstrate that a specific type of input best predicts children’s production frequencies across the domains of surrounding and directed input: speech from other children. Together, these findings expand our perspective beyond dyadic adult-child interactions, supporting the view that child-surrounding speech and especially speech from other children provide important learning opportunities.

## INTRODUCTION

Despite the enormous diversity of languages across the world, children become competent speakers of their native language across all cultures. For decades, research has tried to identify the mechanisms underlying this resilient phenomenon. A considerable amount of this research has been devoted to language input and the question of how it relates to language learning (e.g., Ambridge et al., 2015; Casillas, 2023; Lieven et al., 2009). In particular, dyadic interactions between children and adults have been the primary focus in this line of research. As a result, there is considerable evidence that a specific type of input is strongly associated with language learning: child-directed speech (CDS) (e.g., Golinkoff et al., 2015; Goodman et al., 2008; Rowe, 2012; Spinelli et al., 2017). CDS is often described as a special speech register used by caregivers to address infants and young children and is known to attract infants’ attention more than adult-directed speech (e.g., Fernald, 1985; The ManyBabies Consortium, 2020).

However, most of the research on language input and its role in language development relies on data from children growing up in Western, child-centered cultures (Kidd & Garcia, 2022). In these environments, children are typically involved in dyadic interactions with adults and receive substantial amounts of CDS from an early age onward. This model of interaction, however, is less common in less child-centered cultures, where young children tend to spend their daily lives in multiparty social constellations (Keller, 2022; Lieven, 1994; Ochs & Schieffelin, 1984; Sperry et al., 2019). A substantial line of research has pointed out considerable cross-cultural variation in caregiver-child interactions across human cultures, demonstrating differences in infants’ exposure to linguistic input. In some cultures, for example, children are exposed to smaller amounts of child-directed input and large amounts of child-surrounding input, which is speech among other speakers that is not directed to them (Casillas et al., 2021; Cristia et al., 2019; Shneidman et al., 2009; Shneidman & Goldin-Meadow, 2012). As a result, recent studies have investigated the potential role of child-surrounding input in language acquisition by providing evidence that children can learn novel words from overhearing a conversation (Arunachalam, 2013; Fitch et al., 2020; Floor & Akhtar, 2006). These studies mainly focus on the level of word learning in experimental setups. Studies on children’s ability to learn language from surrounding speech in naturalistic settings are still rare (Foushee & Srinivasan, 2024). Although children as young as 18 months can learn novel words from surrounding interactions (Gampe et al., 2012), research across different cultures has not found any associations between the amount of surrounding speech to which children are exposed and their vocabulary development (Ramírez-Esparza et al., 2014; Shneidman et al., 2013; Shneidman & Goldin-Meadow, 2012; Weisleder & Fernald, 2013).

Cross-cultural perspectives on language acquisition have also begun to explore the role of speech from other children in language learning (Cristia et al., 2023; Loukatou et al., 2022; Scaff et al., 2024; Shneidman & Goldin-Meadow, 2012). In many cultures, children are exposed to large amounts of speech from other children, both in their directed and surrounding input (Cristia et al., 2023; Loukatou et al., 2022; Scaff et al., 2024; Shneidman & Goldin-Meadow, 2012). So far, however, the impact of speech from other children on language acquisition is largely unclear. On the one hand, findings from naturalistic data from Mayan children revealed no relation between directed input from other children at 24 months and the children’s subsequent vocabulary at 35 months (Shneidman & Goldin-Meadow, 2012). On the other hand, speech from other children seems to be of special interest to children. For example, Shipibo-Konibo and Swiss German children between 8–20 months were shown to pay more attention to the surrounding speech of other children compared to the surrounding speech of adults. In addition, Swiss German children were equally attentive to child speech as they were to CDS from adults (Schick et al., 2024). This suggests that to better understand learning in an ecological context, we need to explore the role of different types of input more deeply. Specifically, understanding children’s learning environments requires examining the distributions of child-directed speech and child-surrounding speech in naturalistic settings. Despite the high prevalence of both child speech and surrounding speech in many cultures, to our knowledge, there is only one systematic study by Loukatou et al. (2022) which analyzed the distribution of linguistic patterns in the input from child and adult speakers in both surrounding and directed input. Their findings suggest that the speech of other children shares features with CDS from adults, highlighting its potential role in language acquisition (Loukatou et al., 2022).

Here, we investigate the distributional features of single words in the total input available to children and how they relate to their own speech production. We use naturalistic language data from children growing up in the Shipibo-Konibo community in Peru, an environment where children are typically surrounded by multiple speakers from different age groups and therefore have the possibility to frequently overhear surrounding speech.

We asked whether a) children’s word frequencies are better predicted by their immediate surrounding or directed input, and b) whether within both types of input, child or adult speech is a better predictor. In addition, we tested for age effects to assess whether the predictors change across different age groups.

## MATERIALS AND METHODS

### Shipibo-Konibo Culture

Our data consists of longitudinal recordings of 12 children selected from the Shipibo-Konibo corpus which is part of the ACQDIV data base (Moran, 2016). Shipibo-Konibo is a Panoan language spoken by approximately 20,000 people (Valenzuela, 2003). The Shipibo-Konibo community lives in approximately 130 separated villages along the Ucayali River valley which is part of the Peruvian Amazon in Eastern Peru. With a few exceptions, these villages can only be reached by boat. Related nuclear families largely form villages (Valenzuela, 2003). As in many other Amazonian cultures, child-led alloparenting is very common (Mezzenzana, 2020). From early on, children often play in large groups, with older children taking care of the younger ones and little supervision by adults (De Carvalho Rodrigues Lopes, 2022). In our sample, data collection took place in two villages which are connected through family bonds.

### Data Collection

Data consists of naturalistic daylong video and LENA (Greenwood et al., 2011) recordings in and around the homes of 12 children aged 12–57 months. Recordings started around nine o‘clock in the morning in the children’s homes and ended around six o’clock in the evening. In addition to the LENA recording device, we used a video camera (Zoom Q8 or Sony PXW-Z90V) placed on a tripod to record children’s interactions. Caregivers and family members were encouraged to continue with their daily routine, to move freely and to ignore the position of the camera. Whenever the target child moved to a new location, the camera was placed in a new position and left alone without the recording assistant being constantly present. The recording distance to the target child was approximately between 2–10 meters. Recordings took place every 3.5 months over a period of 1.5 years, resulting in a total of three to five daylong recording sessions per target child. Data collection was carried out in accordance with the ethical guidelines of the University of Zurich and approved by the ethics committee of the University of Zurich (approval nr. 19.6.9). Permission to carry out this research was further obtained from representatives from both villages. All parents gave their written consent to take part in the study and received a small financial compensation for their participation. A detailed overview of the recording sessions per target child can be found in Table 1.

**Table 1.** Overview of number, age and sex of target children and number of observations per target child.

**Target child**	**Age range (months)**	**Sex**	**Number of observations**	**Mean number and range of unigrams across observations**
TC1	12–23	F	4	22 [6–38]
TC2	13–27	M	5	27.2 [12–49]
TC3	13–23	F	4	19.25 [10–33]
TC4	15–22	F	3	17 [3–38]
TC5	23–37	F	5	39.6 [11–56]
TC6	23–37	F	5	70.4 [37–120]
TC7	24–38	F	5	81.8 [64–86]
TC8	23–40	F	4	68.8 [33–109]
TC9	34–48	F	5	70.4 [51–137]
TC10	35–49	M	5	71.2 [53–87]
TC11	41–52	M	4	93.8 [39–119]
TC12	42–57	M	4	73.5 [45–105]

The average recording duration across all recording sessions is 9.1 hours (±0.5). One hour from each daylong recording was manually transcribed using the following sample scheme: Three segments of 10-min at 10 a.m., 1 p.m., and 4 p.m. Four randomly selected segments of 7.5-min before, between and after the periodically selected clips mentioned above. By combining periodical with random sampling, we aimed to adequately capture families’ daily experiences (Casillas, 2023), in documenting both routine behaviors that are typical for some specific periods of the day as well as more variable spontaneous behavior. This resulted in a final data set of 53 hours of transcribed data.

### Data Annotation and Preparation

All utterances from the corpus were manually annotated by two annotators using ELAN (2023) (Version 6.7) coding for the speakers and the addressee/s and their respective ages. Annotators used a combination of different cues to define these factors: the content of the utterance based on the transcription, the context of the utterance based on the visual information of the interaction, and the gaze of participants involved in the interaction. Following this method, each utterance was annotated as either ‘target child-directed’, ‘other child-directed’, ‘child-surrounding’, or ‘unclear’. We defined an utterance as ‘child-surrounding’ when the speech was in overhearable proximity but not directed to the target child. In addition, speakers were categorized as adults or children, with speakers up to 12 years of age being defined as children. In order to test for reliability between annotators, 15% of the corpus were coded by both annotators. Inter-rater reliability was high (Cohen’s kappa = 0.89), suggesting that categories could be identified mostly unambiguously.

**Figure 1.**
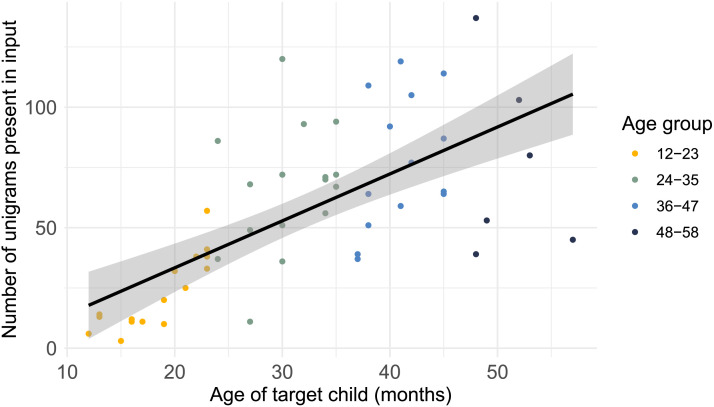
Overview of number of unigrams per target child found in the input of the same day as a function of age in months.

For each transcribed hour of each recording session, the frequencies of each unigram, defined as any unique wordform produced by the target child were extracted using Python (Van Rossum & Drake, 1995) (Version 3.9.13). Additionally, for each unigram present in the speech input of the same recording session, we extracted frequencies in both the surrounding and the directed input from adults and children. Interjections/non-lexicalized back-channels (e.g., mhm) and names were excluded from the analysis. The final dataset consists of a total of 3028 unigrams. Between 3 to 137 unigrams per recording session and target child were found both in the child’s production and in the immediate input (see Table 1). Finally, we grouped our dataset into four twelve-month age groups, from 12–23, 24–35, 36–47, and 48–60 months. No data was excluded, as all children were found to produce at least single-word utterances. Figure 1 shows the number of unigrams per child grouped by age group.

Due to data constraints, utterances coded as ‘other child-directed’ could not be analyzed as a separate category and were therefore added to the broader category of child-surrounding speech. To asses the impact of this decision, we conducted an additional analysis to compare the differences between child-surrounding speech, both including and excluding the category ‘other child-directed speech’. Results suggest no evidence for a difference between child-surrounding input including vs. excluding other child-directed speech (see Supplementary Materials).

### Input Distributions in Shipibo-Konibo

**Figure 2.**
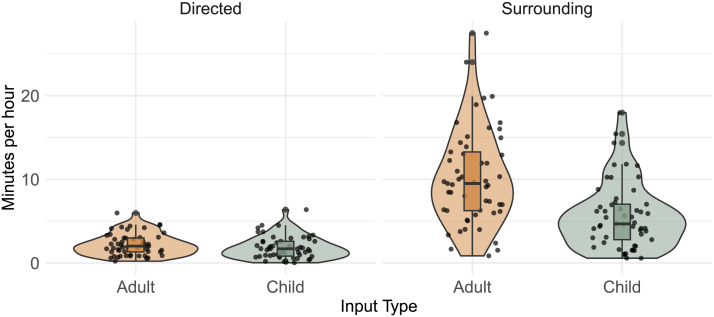
Overview of input distributions of directed and surrounding speech from both adult and child speakers in the Shipibo-Konibo corpus.

Figure 2 shows an overview of the distributions of child-directed and child-surrounding input in our data. On average across all ages, target children were exposed to a mean of 16.3 min (median = 14.65, *SD* = 4.30) of total surrounding input per hour and 3.9 min (median = 4.09, *SD* = 1.08) of directed input per hour. Within directed input, 1.8 min per hour (median = 1.7, *SD* = 0.59) consisted of directed input from other children and 2.1 min per hour (median = 2.02, *SD* = 0.78) of directed input from adult speakers. Within surrounding input, an average of 5.8 min per hour were produced by other children (median = 4.68, *SD* = 3.95) while 10.2 min (median = 9.5, *SD* = 5.69 min) of surrounding input were produced by adults.

Overall, the input rates of CDS are comparable to what has been previously observed through two studies using approximately similar methods estimating input rates of children growing up in small-scale societies (Tseltal Mayan: 3.63 min/hour (Casillas et al., 2020); Yélî Dnye: 3.13 min/hour (Casillas et al., 2021)), while input rates of surrounding speech are lower (Tseltal Mayan: 21.05 min/hour (Casillas et al., 2020); Yélî Dnye: 35.9 min/hour (Casillas et al., 2021)). However, it is important to note that input comparisons should be made with caution as the numbers are very sensitive to definitions of what counts as input (Scaff et al., 2024) and to variability in methods.

### Statistical Analysis

To investigate the relationship between children’s word production and different input types, we fitted three multivariate Bayesian multilevel models through the brms (Bürkner, 2017) interface to Stan (Carpenter et al., 2017) in *R* (Version 4.3.2) (R Core Team, 2022), specifying a Poisson distribution. These models allowed us to simultaneously estimate the effects of predictor variables on multiple dependent variables and the correlations among dependent variables. In order to take potential variability into account, both at the level of the words and target child, an interaction between word and target child was fitted as a random effect in each model. To explore differences across age groups, the interaction was grouped by age group.

Parameters were estimated by running four independent Monte Carlo Markov Chains for 4000 iterations each. We assessed the overall model performance by performing posterior predictive checks, and by calculating a Bayesian *R*^2^-statistic (Gelman et al., 2019). Chain convergence, mixture and stationarity were confirmed by visual inspection of trace plots, and by ensuring that all $\hat{R}$ = 1.00.

**Figure 3.**
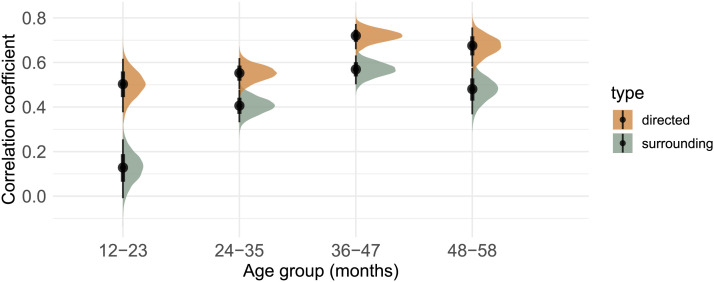
Posterior distributions and means of the correlational coefficients from the mixed-effect model analyzing unigram distributions in the child-directed vs. child-surrounding input in relation to children’s production patterns. Black vertical bars indicate 50% and 90% credible intervals.

**Figure 4.**
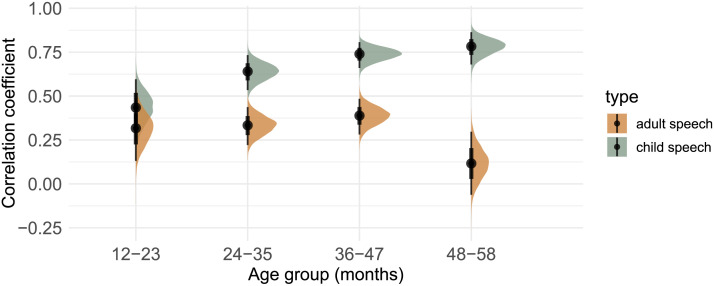
Posterior distributions and means of the correlational coefficients from the mixed-effect model analyzing unigram distributions in the child-directed input from adults vs. children in relation to children’s own production patterns. Black vertical bars indicate 50% and 90% credible intervals.

**Figure 5.**
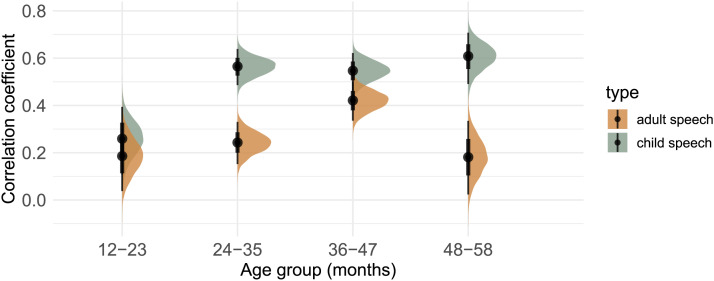
Posterior distributions and means of the correlational coefficients from the mixed-effect model analyzing unigram distributions in the child-surrounding input from adults vs. children in relation to children’s own production patterns. Black vertical bars indicate 50% and 90% credible intervals.

Bayesian methods estimate a posterior distribution, which represents a range of plausible values for each estimated parameter. When reporting the parameter estimates of interest, we provide the posterior mean, the 95% highest density interval (HDI), and the probability of direction (*P*($\hat{\beta }$ > 0)). We consider 95% (or greater) of the HDI (e.g., *P*($\hat{\beta }$ > 0) = 99.9%) in the predicted direction as strong evidence of an effect. We treat 89% (or greater) of the posterior distribution in the predicted direction as weak evidence of an effect, and less than 89% as no evidence. In addition, we plot the posterior distributions for the main parameters of interest (see Figures 3–5). To quantify differences between two analyzed input types in our models, we calculated post-hoc contrasts between the respective input types for each age group in a second step and report these in the main text. The full outcome of all analyses can be found in the Supplementary Materials (see Tables S2–S5). Data and scripts are available on the Open Science Framework repository: https://osf.io/hdsze/.

## RESULTS

### Directed vs. Surrounding Input

We first compared children’s unigram production frequencies with the input distributions of these unigrams in the *total* directed and surrounding input. Results from the first analysis can be found in Figure 3 and Table S2. Our model provides strong evidence for directed input predicting children’s speech production across all age groups (*12–23 months*: mean: 0.50, 95% HDI [0.38, 0.61], *P*($\hat{\beta }$ > 0) = 100%; *24–35 months*: mean: 0.55, 95% HDI [0.48, 0.62], *P*($\hat{\beta }$ > 0) = 100%; *36–47 months*: mean: 0.72, 95% HDI [0.66, 0.77], *P*($\hat{\beta }$ > 0) = 100%; *48–58 months*: mean: 0.67, 95% HDI [0.58, 0.76], *P*($\hat{\beta }$ > 0) = 100%).

When examining surrounding input, our model provides weak evidence for surrounding input predicting children’s speech production in the youngest age group of 12–23 month-olds (mean = 0.10, 95% HDI [−0.03, 0.24], *P*($\hat{\beta }$ > 0) = 93%) and strong evidence for surrounding input predicting children’s speech production starting at age 24 months and older (*24–35 months*: mean: 0.40, 95% HDI [0.33, 0.48], *P*($\hat{\beta }$ > 0) = 100%; *36–47 months*: mean: 0.57, 95% HDI [0.50, 0.63], *P*($\hat{\beta }$ > 0) = 100%; *48–58 months*: mean: 0.48, 95% HDI [0.37, 0.58], *P*($\hat{\beta }$ > 0) = 100%).

Additionally, our model provides strong evidence that compared to child-surrounding input, child-directed input better predicts children’s production patterns across all age groups (*12–23 months*: mean: 0.38, 95% HDI [0.22, 0.53], *P*($\hat{\beta }$ > 0) = 100%; *24–35 months*: mean: 0.14, 95% HDI [0.05, 0.24], *P*($\hat{\beta }$ > 0) = 99.9%; *36–47 months*: mean: 0.15, 95% HDI [0.08, 0.22], *P*($\hat{\beta }$ > 0) = 100%; *48–58 months*: mean: 0.20, 95% HDI [0.08, 0.32], *P*($\hat{\beta }$ > 0) = 99.9%).

### Adult vs. Child Speech: Directed Input

In a second step, we analyzed children’s productions patterns of unigrams comparing directed input patterns from adults with those from other children. Results from the second analysis can be found in Figure 4 and Table S3.

Our model provides strong evidence for child-directed input from adults predicting children’s speech production across age 12–47 months (*12–23 months*: mean: 0.31, 95% HDI [0.13, 0.49], *P*($\hat{\beta }$ > 0) = 100%; *24–35 months*: mean: 0.33, 95% HDI [0.22, 0.44], *P*($\hat{\beta }$ > 0) = 100%; *36–47 months*: mean: 0.39, 95% HDI [0.28, 0.49], *P*($\hat{\beta }$ > 0) = 100%) and weak evidence for the oldest age group of 48–58 months-olds (mean: 0.12, 95% HDI [−0.06, 0.30], *P*($\hat{\beta }$ > 0) = 90%).

When examining child-directed input from children, our model provides strong evidence for this type of input predicting children’s own speech production across all age groups (*12–23 months*: mean: 0.43, 95% HDI [0.25, 0.60], *P*($\hat{\beta }$ > 0) = 100%; *24–35 months*: mean: 0.64, 95% HDI [0.53, 0.73], *P*($\hat{\beta }$ > 0) = 100%; *36–47 months*: mean: 0.74, 95% HDI [0.66, 0.81], *P*($\hat{\beta }$ > 0) = 100%; *48–58 months*: mean: 0.78, 95% HDI [0.68, 0.86], *P*($\hat{\beta }$ > 0) = 100%).

Additionally, our model provides strong evidence that compared to child-directed input from adults, child-directed input from other children better predicts children’s production frequencies for children aged 24 months and older (*24–35 months*: mean: 0.31, 95% HDI [0.15, 0.46], *P*($\hat{\beta }$ > 0) = 100%; *36–47 months*: mean: 0.35, 95% HDI [0.21–0.48], *P*($\hat{\beta }$ > 0) = 100%; *48–58 months*: mean: 0.66, 95% HDI [0.46, 0.87], *P*($\hat{\beta }$ > 0) = 100%). This suggests that directed speech from other children better predicts children’s production patterns compared to child-directed input from adults. Looking at the youngest age group of children aged 12–23 months, we found no evidence for a difference between child-directed input from adults and children (mean = 0.12, 95% HDI [−0.13, 0.36], *P*($\hat{\beta }$ > 0) = 82%).

### Adult vs. Child Speech: Surrounding Input

In a third step, we analyzed children’s productions patterns of unigrams comparing input patterns from surrounding input from adults and children. Results from the third analysis can be found in Figure 5 and Table S4.

Our model provides strong evidence for child-surrounding input from adults predicting children’s speech production across all age groups (*12–23 months*: mean: 0.18, 95% HDI [0.04, 0.33], *P*($\hat{\beta }$ > 0) = 100%; *24–35 months*: mean: 0.24, 95% HDI [0.15, 0.33], *P*($\hat{\beta }$ > 0) = 100%; *36–47 months*: mean: 0.42, 95% HDI [0.34, 0.50], *P*($\hat{\beta }$ > 0) = 100%; *48–58 months*: mean: 0.18, 95% HDI [0.02, 0.33], *P*($\hat{\beta }$ > 0) = 98.8%).

When examining child-surrounding input from children, our model again provides strong evidence for this type of input predicting children’s own speech production across all age groups (*12–23 months*: mean: 0.26, 95% HDI [0.11, 0.39], *P*($\hat{\beta }$ > 0) = 100%; *24–35 months*: mean: 0.56, 95% HDI [0.49, 0.64], *P*($\hat{\beta }$ > 0) = 100%; *36–47 months*: mean: 0.55, 95% HDI [0.46, 0.62], *P*($\hat{\beta }$ > 0) = 100%; 48–58 months: mean: 0.61, 95% HDI [0.49, 0.71], *P*($\hat{\beta }$ > 0) = 100%).

Additionally, our model provides strong evidence that compared to child-surrounding input from adults, child-surrounding input from other children better predicts children’s production patterns for children aged 24 months and older (*24–35 months*: mean: 0.32, 95% HDI [0.22, 0.43], *P*($\hat{\beta }$ > 0) = 100%; *36–47 months*: mean: 0.13, 95% HDI [0.01, 0.24], *P*($\hat{\beta }$ > 0) = 98.4%; *48–58 months*: mean: 0.43, 95% HDI [0.25, 0.60], *P*($\hat{\beta }$ > 0) = 100%). This suggests that surrounding speech from other children better predicts children’s production patterns compared to surrounding input from adults. Looking at the youngest age group of 12–23 month olds, we found no evidence for a difference between the two types of input in predicting children’s speech patterns (mean: 0.07, 95% HDI [−0.10, 0.24], *P*($\hat{\beta }$ > 0) = 80%).

## DISCUSSION

We provided evidence that children’s word production frequencies in Shipibo-Konibo are best predicted by their total directed input across all age groups. Despite the overall lower amounts of directed input and larger amounts of surrounding input children in our sample are exposed to, overall directed input is the best predictor of their production patterns. Interestingly, we also find that at age 2;0 and later, surrounding input is a reliable predictor of children’s production patterns. So far, prior studies analyzing quantitative features of directed and surrounding input have not found any correlations between the amount of surrounding speech children are exposed to and their vocabulary development (Ramírez-Esparza et al., 2014; Shneidman et al., 2013; Shneidman & Goldin-Meadow, 2012; Weisleder & Fernald, 2013). Our study demonstrates that despite these findings, the frequency distributions of unigrams in the surrounding input are remarkably similar to children’s own production frequencies, indicating that this source of input may provide valuable learning opportunities. This result is consistent with the finding of considerable qualitative overlap between child-surrounding and directed speech of an English-learning child, looking at qualitative features that make language learnable, such as referential gestures or references of the here and now (Foushee, 2020).

Looking at directed input only, our results demonstrate that at the age of 2;0 and later, directed speech from other children is clearly the best predictor of children’s production patterns. Our results suggest that, as Shipibo-Konibo children grow older, their speech production becomes more similar to directed input from other children around them and less similar to directed input from adults. Building up on previous work (Cristia et al., 2023; Loukatou et al., 2022; Scaff et al., 2024; Shneidman & Goldin-Meadow, 2012), these results add further insights to the role of speech from other children in language development and support the hypothesis that this type of input provides an eligible source of language learning for the child. This is supported by the fact that child speech shares several acoustic and structural characteristics with the well-studied CDS from adults (Lee et al., 1999; Loukatou et al., 2022; Potamianos & Narayanan, 2007), provides more relevant topics, and relates more to children’s immediate experiences than adult speech. Additionally, the social function of peers and older children in child development is well established (Bailey et al., 1993; Rubin et al., 2011; Zmyj et al., 2012; Zmyj & Seehagen, 2013) and may further facilitate language learning from peers.

Looking at surrounding input only, we again find strong evidence for surrounding speech from other children being a better predictor of children’s production frequencies at age 2;0 and later, compared to surrounding speech from adults. Results again serve as a strong indicator for speech from other children playing an important role. They are consistent with prior experimental work which has demonstrated that children pay more attention towards surrounding speech from other children (compared to surrounding speech from adults) providing further evidence supporting the previously mentioned hypothesis on the possibly underestimated role of speech from other children (Schick et al., 2024). Assuming that children do learn from surrounding input, both findings suggest that surrounding speech from other children provides a better source for learning compared to surrounding adult speech.

Together, our results demonstrate that both directed and surrounding speech are diverse sources of input (Foushee, 2020) including speech from various speakers of different ages, providing children with diverse opportunities to learn language. Although these findings do not provide direct evidence of learning, they have important implications for learning. Our interpretation builds on previous research suggesting that infants’ attention is sensitive to variation in the input. For example, research has shown that CDS attracts the attention of infants’ more than adult-directed speech (e.g., Fernald, 1985; The ManyBabies Consortium, 2020; Werker & McLeod, 1989). An effect that is associated to play an important role in eliciting and maintaining children’s attention and supporting language acquisition (Spinelli et al., 2017). Further research has shown that child-surrounding speech from children captures infant’s attention more successfully compared to child-surrounding speech from adults (Schick et al., 2024). This further supports the idea that infant’s attention is responsive to different types of input. Additionally, evidence suggests that infants preferentially focus on input that aligns with a learnable level of complexity (e.g., Foushee et al., 2023; Gerken et al., 2011; Kidd et al., 2014). Building on these findings, we assume that a high similarity between children’s production and their immediate linguistic environment suggests that they may greatly benefit from this source of input for language learning. Looking at our results regarding input from other children, we propose that particularly language learning from older peers represents a promising area for future research.

The question remains whether we find comparable patterns in children growing up in a different cultural environment with a) lower amounts of surrounding speech and b) lower amounts of speech from other children compared to the Shipibo-Konibo corpus used in this study. Additionally, examining the distribution of more complex linguistic patterns in later stages of development as tested in this study, could offer further valuable insights into this topic.

Finally, building on the growing evidence from experimental overhearing studies (Arunachalam, 2013; Fitch et al., 2020; Floor & Akhtar, 2006; Foushee & Srinivasan, 2024), we offer an new perspective on this topic by examining production patterns of naturalistic language data. We hope that this approach contributes to a better understanding of how surrounding speech, especially from children, can serve as an important source of input to the language learning child. Future studies could benefit from exploring cross-cultural research that extends beyond urban and Western populations, which would help provide a more comprehensive understanding of language acquisition and the impact of diverse cultural environments on young children’s linguistic experiences.

## Acknowledgments

We are very grateful to all the caregivers and infants in Peru who participated in our study. We especially thank Edelvina Cumapa Campos and Noely Damaris Barbaran Campos for their invaluable help with data collection in Peru.

## Funding Information

This work was funded by the NCCR Evolving Language, SwCSS NSF Agreement No. 51NF40_180888.

## Author Contributions

JS: Conceptualization; Data curation; Formal analysis; Investigation; Methodology; Writing—Original draft; Writing—Review & editing. SS: Conceptualization; Funding acquisition; Writing—Review & editing.

## Data Availability Statement

The data that support the findings of this study are available on the OSF repository: https://osf.io/hdsze/.

## Supplementary Material


