# Supplementary material for: Children Learn Best From Their Peers: The Crucial Role of Input From Other Children in Language Development
Source: Open Mind (Camb). 2025 Apr 29;9:665–76. doi: 10.1162/opmi_a_00198 (PMC12058327; doi:10.1162/opmi_a_00198)
Supplement: Supplementary file 1 [file opmi-09-665-s001.pdf]

Supplementary Information for: ”Children learn best from their peers: The crucial role of input from other children in language development”

Johanna Schick
Institute for the Interdisciplinary Study of Language Evolution, University of Zurich
johanna.schick@uzh.ch

Sabine Stoll
Institute for the Interdisciplinary Study of Language Evolution, University of Zurich
sabine.stoll@uzh.ch

S1: Other child-directed speech

Figure S1 shows an overview of the distributions of three subcategories of the broader category of of child-surrounding speech: child-surrounding speech from adults, other child-directed speech from adults (OCDS) and child-surrounding speech from children. On average across all ages, target children were exposed to a mean of 1.7 min (median = 1.49, SD = 1.53) of OCDS per hour. In order to explore the role of OCDS in child-surrounding input from adults, we fitted a Bayesian multivariate mixed-effect model to analyze whether there is a difference in child-surrounding input from adults predicting target children's unigram frequencies comparing both child-surrounding input including vs. excluding OCDS. We fitted a model with the same model settings as in the main analysis. Results can be found in Figure S2 and Table S1.

We used post-hoc contrasts to quantify differences between the two analyzed input types in each age group and found no evidence for a difference between surrounding input from adults including vs. excluding OCDS in predicting children's unigram frequencies across the three youngest age groups (*12-23 months*: mean: 0.03, 95% HDI [-0.02, 0.07]; *24-35 months*: mean: 0.03, 95% HDI [-0.01, 0.05]; *36-47 months*: mean: 0.04, 95% HDI [-0.02, 0.06]). In the oldest age group, we found weak evidence for surrounding input from adults including OCDS better predicting children's unigram frequencies compared to surrounding input from adults excluding OCDS (*48-58 months*: mean: 0.07, 95% HDI [-0.01, 0.16]).

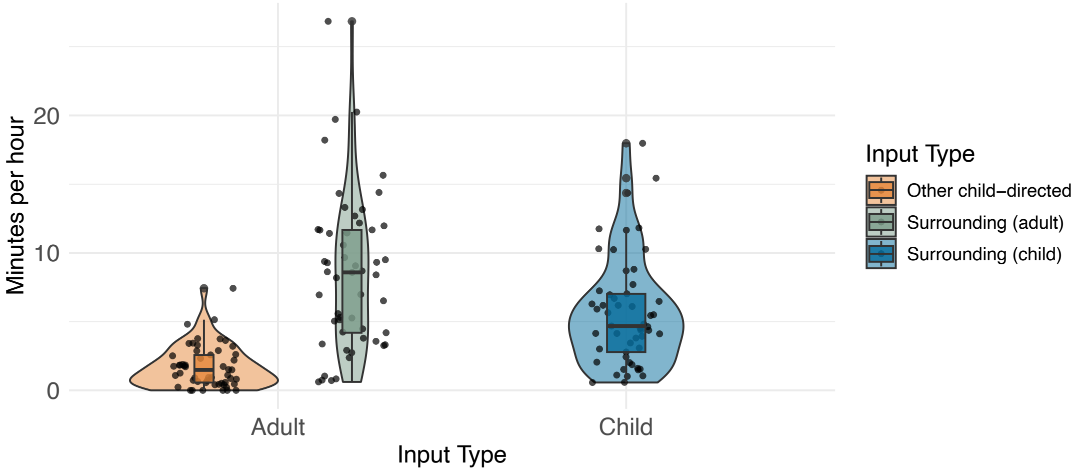

Figure S1: Overview of input distributions of the category ‚child-surrounding speech‘ in the Shipibo-Konibo corpus.

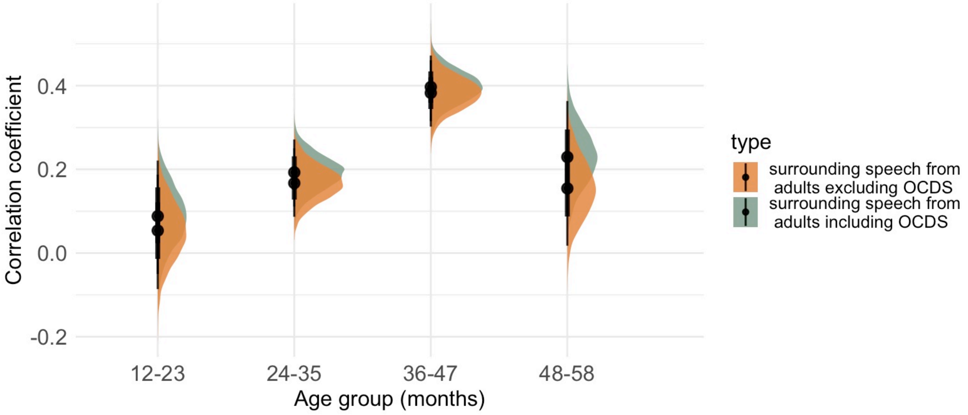

Figure S2: Posterior distributions and means of the correlational coefficients from the mixed-effect model analyzing unigram distributions in the child-surrounding input from adults, comparing OCDS-included with OCDS-excluded child-surrounding input in relation to children's own production patterns. Horizontal bars indicate 50% and 90% credible intervals

	Parameter	Estimate	Est.Error	Lower 95% CI	Higher 95% CI
12-23 months	OCDS included	0.10	0.07	-0.03	0.23
	OCDS excluded	0.07	0.07	-0.06	0.20
24-35 months	OCDS included	0.20	0.04	0.11	0.28
	OCDS excluded	0.17	0.04	0.09	0.25
36-47 months	OCDS included	0.41	0.04	0.37	0.52
	OCDS excluded	0.39	0.04	0.32	0.48
48-60 months	OCDS included	0.22	0.07	0.08	0.34
	OCDS excluded	0.15	0.07	0.01	0.28

Table S1: Posterior distributions of correlational coefficients from the multivariate Bayesian mixed-effect model analyzing child-surrounding input from adults comparing OCDS-included with OCDS-excluded surrounding input from adults in relation to children's own production frequencies. CI = credible interval.

S2: Model summaries

Model 1

	Parameter	Estimate	Est.Error	Lower 95% CI	Higher 95% CI
12-23 months	surrounding	0.13	0.07	-0.01	0.25
	directed	0.50	0.06	0.38	0.62
24-35 months	surrounding	0.41	0.04	0.33	0.48
	directed	0.55	0.04	0.48	0.62
36-47 months	surrounding	0.57	0.03	0.50	0.63
	directed	0.72	0.03	0.66	0.77
48-60 months	surrounding	0.48	0.05	0.37	0.58
	directed	0.67	0.05	0.58	0.76

Table S2: Posterior distributions of correlational coefficients from the multivariate Bayesian mixed-effect model analyzing child-directed vs. child-surrounding input frequencies in relation to children's own word production frequencies. CI = credible interval.

Model 2

	Parameter	Estimate	Est.Error	Lower 95% CI	Higher 95% CI
12-23 months	directed: adult	0.31	0.09	0.13	0.49
	directed: child	0.43	0.09	0.25	0.60
24-35 months	directed: adult	0.33	0.06	0.22	0.44
	directed: child	0.64	0.05	0.53	0.73
36-47 months	directed: adult	0.39	0.05	0.28	0.48
	directed: child	0.74	0.04	0.66	0.81
48-60 months	directed: adult	0.12	0.09	-0.06	0.30
	directed: child	0.78	0.05	0.68	0.86

Table S3: Posterior distributions of correlational coefficients from the multivariate Bayesian mixed-effect model analyzing child-directed input frequencies from children vs. child-directed input frequencies from adults in relation to children's own word production frequencies. CI = credible interval.

Model 3

	Parameter	Estimate	Est.Error	Lower 95% CI	Higher 95% CI
12-23 months	surrounding: adult	0.18	0.07	0.04	0.33
	surrounding: child	0.26	0.07	0.11	0.39
24-35 months	surrounding: adult	0.24	0.05	0.15	0.33
	surrounding: child	0.56	0.04	0.49	0.64
36-47 months	surrounding: adult	0.42	0.04	0.34	0.50
	surrounding: child	0.55	0.04	0.46	0.62
48-60 months	surrounding: adult	0.18	0.08	0.02	0.33
	surrounding: child	0.61	0.05	0.49	0.71

Table S4: Posterior distributions of correlational coefficients from the multivariate Bayesian mixed-effect model analyzing child-surrounding input frequencies from children vs. child-surrounding input frequencies from adults in relation to children's own word production frequencies. CI = credible interval.
